# Controlling ammonia in aquatic environments: a critical review linking monitoring, prevention, and recovery

**DOI:** 10.1007/s10661-026-16001-w

**Published:** 2026-10-21

**Authors:** Jiří Lindovský, Renata Komendová

**Affiliations:** Faculty of Chemistry, Brno University of Technology, Purkyňova 118/464, Brno, 61200 Czech Republic

**Keywords:** Ammonia removal, Environmental regulation, Wastewater treatment, Separation, Pollution

## Abstract

Ammonia is a key inorganic nitrogen species connecting aquatic chemistry, ecosystem toxicity, and atmospheric particulate formation. In water, environmental risk is not governed solely by total ammonia nitrogen (TAN), but by the speciation equilibrium between NH$_3$ and $\text {NH}_{4}^{\,\,+}$, controlled by pH, temperature, and hydrological variability. Increasing nitrogen loads from wastewater effluents, anaerobic digestion residues, and agricultural activities generate short-term concentration peaks that are difficult to capture by conventional monitoring and complicate regulatory management. At the same time, tightening discharge limits and circular-economy policies require strategies that both mitigate environmental impacts and enable nutrient recovery. However, ammonia is still typically addressed separately in environmental chemistry, agricultural management, and wastewater engineering, preventing coordinated mitigation. Here, we review environmental ammonia in aquatic systems using an integrated chemical–environmental–technological perspective. The major points are the following: (i) chemical speciation governs toxicity, volatilization, and environmental transport more strongly than total concentration alone; (ii) analytical monitoring approaches, from colorimetric and ion-selective electrode measurements to sensors and passive sampling techniques, are essential for interpreting environmental exposure and guiding regulation; (iii) upstream measures, including controlled-release fertilizers and urease inhibitors, reduce emission peaks at the source; (iv) physicochemical and biological treatment processes such as air stripping, membrane contactors, adsorption, and nitrification enable ammonia removal and recovery from wastewater and digestate; and (v) recovered ammonia can serve as a secondary nitrogen resource within circular nutrient management. Integrating monitoring, prevention, treatment, and reuse provides a framework for data-driven ammonia control across environmental and engineered water systems.

## Introduction

Ammonia is among the problematic pollutants of the aquatic environment. In the environment, it poses a significant risk—in the form of free ammonia (NH$_3$), which is toxic to aquatic organisms and contributes to eutrophication of surface waters, and its presence in watercourses is often associated with reduced biodiversity (Jin et al., 2025). From an environmental point of view, ammonia represents an undesirable contaminant, whereas in industry and agriculture, it is, at the same time, a highly valued raw material. The production of ammonia by the Haber–Bosch process is one of the most energy-intensive industrial operations in the world, accounting for approximately 1–2% of global energy consumption (Chavando, 2023; Kyriakou et al., 2020). This means that every loss of TAN to the environment is not only an ecological problem but also an economic loss.

The sources of total ammonia nitrogen (TAN) in liquid waste streams are diverse. In addition to municipal wastewater, where it is mainly produced by the decomposition of proteins and urea, significant contributions come from industrial facilities—especially the food-processing, chemical, and fertilizer industries. A specific and increasingly important group of sources is represented by biogas plants, where ammonia in high concentrations passes into the digestate after anaerobic digestion. Based on data from biogas plants, the average TAN concentration is typically on the order of grams per liter, depending on the type of substrate processed in the anaerobic digestion system. Other sources include slurry and manure from animal production, as well as concentrated industrial wastewater streams (Deng et al., 2021). All of these flows have one thing in common: they contain TAN at concentrations that require its effective removal or separation.

**Fig. 1:**
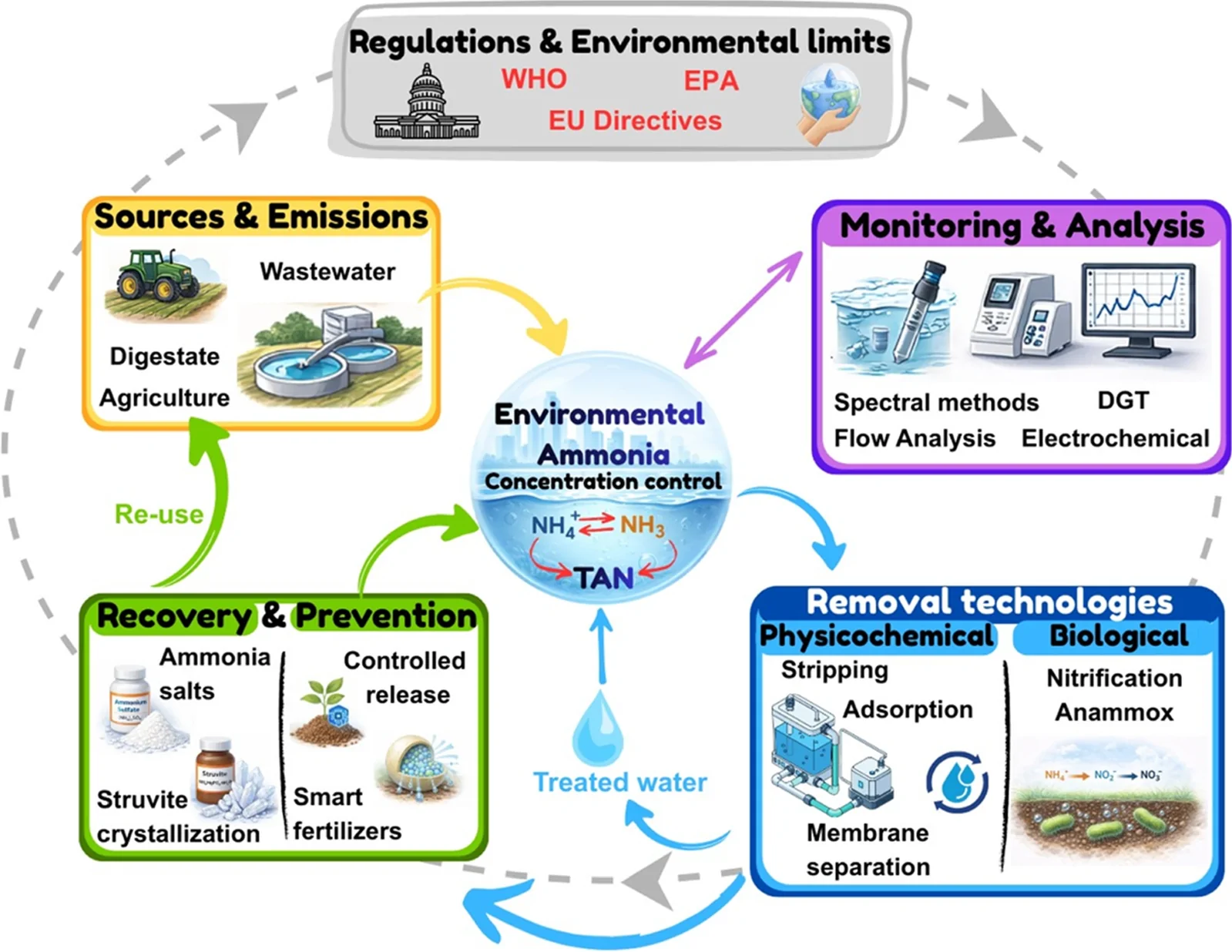
Integrated framework for environmental ammonia management linking monitoring, regulation, removal, and resource recovery

The legislation related to the removal of TAN from wastewater has gradually been tightened. In Europe, the key frameworks include the Urban Waste Water Treatment Directive (UWWTD), the Industrial Emissions Directive (IED), and the related Best Available Techniques (BAT) Conclusions. These documents set strict limits for nitrogen discharge, thereby creating pressure to implement more efficient treatment technologies (Bjerg et al., 2023; Parliament, 2024). In the Czech Republic, for example, emission limits are defined in the Government Regulation No. 401/2015 Coll., which specifies the permissible concentrations of ammoniacal nitrogen for various types of pollution sources. This Government Regulation can be seen as the national implementation of the broader EU legislative framework for water protection and pollution control, particularly in terms of setting indicators and limits for wastewater discharge and water quality assessment. In the European context, it builds primarily on the Water Framework Directive (2000/60/EC) and related regulations on environmental quality standards and priority substances (2008/105/EC and amendment 2013/39/EU), as well as the Industrial Emissions Directive (2010/75/EU). Other older water directives are also mentioned in the references  Česko (2015). Similar trends can be observed in other European countries, whereas in non-European contexts (for example, in the USA or China), the issue of ammonia pollution is not always addressed with comparable strictness, and the regulations are often focused more on total nitrogen or on other pollutants (Lu et al., 2016).

Effective ammonia management requires more than efficient removal technologies or accurate environmental monitoring alone. Ammonia is a highly dynamic pollutant whose environmental impact is governed by interactions between chemical speciation, hydrological conditions, regulatory requirements, monitoring strategies, and treatment technologies. Consequently, decisions regarding monitoring, pollution prevention, process selection, and nutrient recovery cannot be made independently. Nevertheless, these aspects are still predominantly investigated within separate scientific disciplines, with environmental studies focusing on occurrence and ecological impacts, whereas engineering research primarily emphasizes treatment efficiency and process optimization. This disciplinary separation limits the translation of environmental observations into practical management strategies and hinders the development of integrated ammonia management frameworks (Fig. 1).

This review presents ammonia as a central component of an integrated environmental management system rather than as an isolated wastewater pollutant or treatment target. Instead of providing another technology-oriented review, it integrates current knowledge on ammonia occurrence, chemistry, monitoring, legislation, prevention, treatment, and resource recovery into a unified systems-level framework. A key contribution of this review is the integration of disciplines that are traditionally considered separately, including environmental chemistry, environmental monitoring, regulatory policy, agriculture, wastewater engineering, and nutrient recovery. By connecting these fields, the review demonstrates how monitoring, legislation, prevention, treatment technologies, and resource recovery collectively support effective ammonia management across aquatic and engineered systems. In addition, it highlights emerging directions, including resource recovery, digital monitoring, and sustainable wastewater management, to provide a broader perspective on future ammonia management. To the best of our knowledge, no previous review has combined these aspects within a single environmental management framework to support both environmental assessment and engineering decision-making.

## Ammonia in the environment

### Legislation

Ammonia is one of the common pollutants in wastewater, and its high concentrations can easily cause environmental contamination. The most important negative effects of high TAN concentrations are toxicity for aquatic animals, drinking water and groundwater contamination, and reducing oxygen in water/soil caused by the nitrification process (Camargo & Alonso, 2006). Sources of ammonia-contaminated water and liquid wastes come mainly from agriculture. According to the European Environment Agency, ammonia emissions from agriculture in Europe accounted for approximately 90% of total ammonia emissions between 2011 and 2022 (Eurostat, 2024). In agriculture, the majority of ammonia emissions (78%) originate from the management of livestock manure, with 49% specifically linked to the use of natural fertilizers. The remaining 22% is associated with the application of mineral nitrogen fertilizers on agricultural land. Emissions from non-agricultural sectors are minimal: combustion processes outside agriculture account for 2.5%, road transport contributes 1.5%, and industrial combustion processes 1.2% (Murawska & Prus, 2021). In terms of European legislation, there are guidelines that establish TAN concentrations in different sectors and describe the handling of these highly concentrated wastes. For example, Council Directive 91/676/EEC limits nitrogen compounds (such as ammonia) to reduce water pollution caused by nitrates from agricultural sources. In addition to regulating the handling of ammonia-containing waste, the directive sets an annual nitrogen application limit of 170 kg ha$^{-1}$ (Council of the European Communities, 1991b). The NEC Directive (EU) 2016/2284 sets binding targets for reducing ammonia emissions across the EU, primarily from agriculture. Member States must reduce national ammonia emissions by 6% for the period 2020–2029 and by 16% from 2030 onward, compared to 2005 levels. Measures include improved manure storage, low-emission application techniques, and optimized fertilization and feeding strategies. Although aimed at improving air quality, the directive also helps reduce nitrogen pollution in water bodies (Parliament, 2016). In the context of wastewater, the Urban Waste Water Treatment Directive (91/271/EEC, UWWTD) plays a key role. Although it does not directly regulate TAN, it requires the removal of nutrients (nitrogen and phosphorus) in sensitive areas to prevent eutrophication. Compliance with the total nitrogen limits set by the directive therefore implicitly requires efficient ammonia removal, typically through nitrification–denitrification processes (Council of the European Communities, 1991a). Industrial and agricultural point sources of ammonia are also regulated under the Industrial Emissions Directive (2010/75/EU, IED). This directive establishes an integrated permitting system based on Best Available Techniques (BAT) and sets binding emission levels (BAT-AELs) for large-scale industrial installations. Several BAT reference documents (BREF) specifically mention ammonia, for example, in intensive livestock production or in fertilizer and chemical manufacturing (Parliament, 2010). In addition, the Water Framework Directive (2000/60/EC, WFD) sets the overall objective of achieving good ecological and chemical status of surface and groundwater (Parliament, 2000). Ammonia is considered a key pollutant affecting ecological quality, and national monitoring programs must assess its concentration as part of water body classification. Speaking about drinking water, Directive (EU) 2020/2184 marks ammonium as an indicator parameter, and its parametric value is 0.5 mg l$^{-1}$. It is up to member states to evaluate the risk to the human health and adjust the values if necessary (Parliament, 2020). The WHO takes a similar approach, and according to the Guidelines for Drinking-water Quality, the recommended concentrations are 1.5 mg l$^{-1}$ for ammonia (odor threshold) and 35 mg l$^{-1}$ for the ammonium cation (taste threshold), while NH$_3$ is 190 times more toxic than NH$_4^+$ (World Health Organization, 2011). Meanwhile, for humans, there is no strict limitation on TAN in water; what is crucial is the risk posed by the TAN concentration to aquatic life.

The regulation of total ammonia nitrogen in freshwater systems is strongly dependent on pH and temperature, as the toxicity is governed by the fraction of unionized ammonia NH$_3$. The U.S. Environmental Protection Agency (U.S. EPA) has issued recommended criteria indicating that, under standard conditions (pH 7.0; 20 $^\circ$C), acute exposure (1 h) should not exceed 17 mg TAN l$^{-1}$, while chronic exposure should not exceed a 30-day rolling average of 1.9 mg TAN l$^{-1}$ (Agency, U. S. E. P., 2013). Long-term national monitoring data suggest that exceedances of recommended TAN criteria in the US surface waters are relatively rare, although short-term concentration peaks may be underestimated due to low sampling frequency. TAN is not regulated in drinking water in the USA, by WHO, or in the EU; however, the EU designates ammonium as an indicator parameter with a threshold of 0.5 mg NH$_4^+$ l$^{-1}$ (Edwards et al., 2024). Table 1 summarizes the recommended concentration limits for total TAN and additional information.

**Table 1:** Regulatory and guideline limits for total ammonia nitrogen (TAN), ammonia, and ammonium in water

Source	Metric	Original value	Converted	Notes
			(mg TAN l$$^{-1}$$)	
U.S. EPA	Acute criterion (1 h)	17.0 mg TAN l$^{-1}$	17.00	Fresh water
U.S. EPA	Chronic criterion	1.9 mg TAN l$^{-1}$	1.90	Fresh water
EU Directive (EU) 2020/2184	$\text {NH}_{4}^{\,\,+}$ indicator parameter	0.5 mg $\text {NH}_{4}^{\,\,+}$ l$^{-1}$	0.39	Drinking water
WHO Guidelines	NH$_3$ odor threshold	1.5 mg NH$_3$ l$^{-1}$	1.24	Drinking water
WHO Guidelines	$\text {NH}_{4}^{\,\,+}$ taste threshold	35.0 mg $\text {NH}_{4}^{\,\,+}$ l$^{-1}$	27.20	Drinking water

In China, surface waters are classified into five categories (I–V) according to the national environmental quality standard GB 3838–2002, with classification based on the intended water use and the required level of environmental protection. Category I includes headwater streams and national nature reserves, while Category II applies to primary drinking water protection zones and habitats of rare aquatic species. Category III covers secondary drinking water protection zones, recreational waters, and fisheries, whereas Categories IV and V are intended for industrial and agricultural water use, respectively. The corresponding ammonia nitrogen (NH$_3$-N) limits increase progressively from 0.15 mg l$^{-1}$ for Category I to 2.0 mg l$^{-1}$ for Category V (0.15, 0.5, 1.0, 1.5, and 2.0 mg l$^{-1}$, respectively), providing a clear regulatory framework that links water quality requirements to the designated environmental function of each water body (Ministry of Ecology and Environment of the People’s Republic of China, 2002).

Unlike regulatory systems based on fixed national concentration limits, Australia and New Zealand apply a risk-based framework in which default ammonia trigger values are adjusted according to ecosystem type and regional environmental characteristics. Across different Australian regions and New Zealand, the recommended trigger values range from approximately 1 to 100 µg NH$_4^+$ l$^{-1}$, representing a two-order-of-magnitude variation. In addition, the guidelines establish ecotoxicological trigger values for total ammonia (NH$_3$-N at pH 8) corresponding to different levels of species protection. For freshwater ecosystems, these values range from 320 µg NH$_3$-N l$^{-1}$ for 99% species protection to 2300 µg NH$_3$-N l$^{-1}$ for 80% protection. This approach demonstrates that water quality management is based not only on fixed concentration thresholds but also on site-specific environmental conditions and clearly defined ecological protection objectives (Australian and New Zealand Environment and Conservation Council, 2000; Zhang et al., 2022).

Latin America represents a markedly different regulatory landscape, as no harmonized regional framework for reactive nitrogen management has been established. Existing legislation is primarily developed at the national level and generally focuses on individual environmental sectors rather than adopting an integrated approach to nitrogen management. Recent analyses identify fragmented legislation, limited institutional capacity, and insufficient policy integration as the main barriers to effective nitrogen governance across the region. Consequently, despite increasing pressures from agricultural intensification, rapid urbanization, biomass burning, and inadequate wastewater treatment, comprehensive strategies for managing reactive nitrogen remain underdeveloped in most Latin American countries (Cunha-Zeri & Ometto, 2021).

These examples demonstrate that approaches to ammonia regulation differ substantially worldwide. The European Union relies on harmonized legally binding legislation, China applies nationally standardized function-based water quality criteria, Australia and New Zealand use adaptive risk-based trigger values tailored to ecosystem characteristics, whereas Latin America is characterized by fragmented national policies and the absence of an integrated regional nitrogen management framework.

### Factors influencing the occurrence of ammonia in the environment

The occurrence of ammonia and the distribution of its chemical forms vary dynamically over time, influenced by hydrological conditions, temperature, and biological activity. Consequently, ammonia generation is governed more by seasonal extremes and short-term events than by long-term average conditions (Edwards et al., 2024).

From a hydrological perspective, seasonally reduced streamflows can lead to localized increases in ammonia concentrations in affected areas. In contrast, rainfall events and storms, in combination with topographic factors, can accelerate nutrient transport to soils and surface waters, resulting in short-term concentration peaks (Luthy et al., 2015). Temperature also plays a crucial role, as low winter temperatures reduce the activity of nitrifying bacteria in wastewater treatment plants, leading to elevated ammonia concentrations (Johnston et al., 2019). Seasonal increases in pH and temperature also shift the ammonia equilibrium toward the unionized form NH$_3$, which is more toxic to aquatic organisms and more prone to volatilization into the atmosphere. Consequently, identical TAN concentrations can pose markedly different environmental risks depending on the period of the year (Emerson et al., 1975).

Atmospheric NH$_3$ exhibits a highly localized and episodic distribution due to its short atmospheric lifetime, resulting in concentration hotspots over intensively farmed regions. Large-scale syntheses of measurement, satellite, and model-based studies indicate that elevated NH$_3$ concentrations are typically confined to agricultural areas such as the North China Plain, the US Midwest, and the Indo-Gangetic Plain. Reported hotspot levels reach tens of ppbv, with monthly average concentrations ranging from approximately 20 to 74 ppbv depending on the region (Nair & Yu, 2020).

Agriculture dominates the global NH$_3$ emission budget, accounting for approximately 81% of total emissions. Atmospheric NH$_3$ is also a major precursor of secondary particulate matter formation, particularly ammonium nitrate, contributing about 50% of PM$_{2.5}$ in the European Union and approximately 30% in the USA. Consequently, mitigation of NH$_3$ emissions from agricultural sources represents an efficient strategy for improving air quality and achieving significant public health benefits (Wyer et al., 2022).

While local-scale effects are discussed above, at the regional and national scales, ammonia is primarily addressed through emission inventories and sector-based mitigation strategies, with agriculture dominating NH$_3$ emissions in many regions (Jiang et al., 2024). At these scales, diffuse sources largely determine long-term average concentrations, whereas local technological interventions represent a controllable component within a broader mitigation portfolio. At the global scale, ammonia is recognized as a key driver of nitrogen-cycle disruption and a multi-media pollutant linking air, water, and soil compartments. Ongoing climate warming is expected to further enhance ammonia variability by increasing volatilization and intensifying hydrological extremes, thereby reinforcing the need for targeted, data-driven control at local hotspots (Sutton & UNEP, 2013).

Collectively, these studies demonstrate that ammonia occurrence is governed primarily by temporal variability rather than by average annual concentrations. Consequently, conventional periodic monitoring often fails to capture environmentally relevant short-term concentration peaks generated by rainfall events, seasonal agricultural activities, or wastewater treatment disturbances. This finding emphasizes that improvements in ammonia management require not only efficient treatment technologies but also monitoring strategies capable of resolving transient pollution events.

## Ammonia control and management

Ammonia must be removed from a wide range of sources, including municipal and industrial wastewater, liquid fertilizers generated by agricultural activities, and sewage sludge. At the same time, increasingly stringent regulations are being imposed on the discharge of pollutants such as ammonia, driving the need for more effective treatment solutions. Consequently, intensive research and development efforts are focused on separation technologies that offer lower operating costs while efficiently meeting emission limits. A broad spectrum of physicochemical processes is being explored, including membrane-based technologies, absorption and desorption processes, adsorption, and electrochemical methods.

### Ammonia distribution

Efficient separation of ammonia relies on understanding its physicochemical properties. Theoretical calculations describing the distribution of ammonia between the gaseous and liquid phases are based on Henry’s law:

1 $$\begin{aligned} H = \frac{c_A}{p_A} \end{aligned}$$

Historically, it has been referred to as a constant; however, it is influenced by many parameters. The most important factor affecting Henry’s constant is temperature, and this dependence is described by the van’t Hoff equation:

2 $$\begin{aligned} \frac{\textrm{d}\ln H}{\textrm{d}\left( 1/T\right) } = -\frac{\Delta h_{\textrm{sol}}}{R} \end{aligned}$$

Integration of Eq. 2 allows conversion between standard Henry’s constant $H_{\textrm{0}}$ (for $T_{\textrm{0}}$ = 298.15 K) and the value at the desired temperature:

3 $$\begin{aligned} H = H^{0} \cdot \exp \!\left( -\frac{\Delta h_{\textrm{sol}}}{R} \left( \frac{1}{T} - \frac{1}{T^{0}} \right) \right) \end{aligned}$$

In the literature, many forms of Henry’s constant can be found, differing in their method of calculation. However, for the purpose of obtaining information about gas solubility, the most convenient form of Henry’s constant is expressed in mol m$^{-3}$ Pa$^{-1}$. For example, Sander (2015) reported a Henry’s constant for ammonia of 60.2 $\mathrm {mol\,dm^{-3}\,atm^{-1}}$, which corresponds to 0.594 mol m$^{-3}$ Pa$^{-1}$ (at $T_{\textrm{0}}$= 298.15 K) and follows the van’t Hoff equation within the temperature range of 273–348 K. After conversion to verified temperatures, this value approximately agrees with the results of Dasgupta and Dong (1986), who, based on thermodynamic calculations, determined that within the temperature range 278.8–290.6 K, Henry’s constant can be expressed as follows:

4 $$\begin{aligned} \ln H = \frac{4092}{T} - 9.7 \; [\mathrm {mol\,dm^{-3}\,atm^{-1}}] \end{aligned}$$

**Fig. 2:**
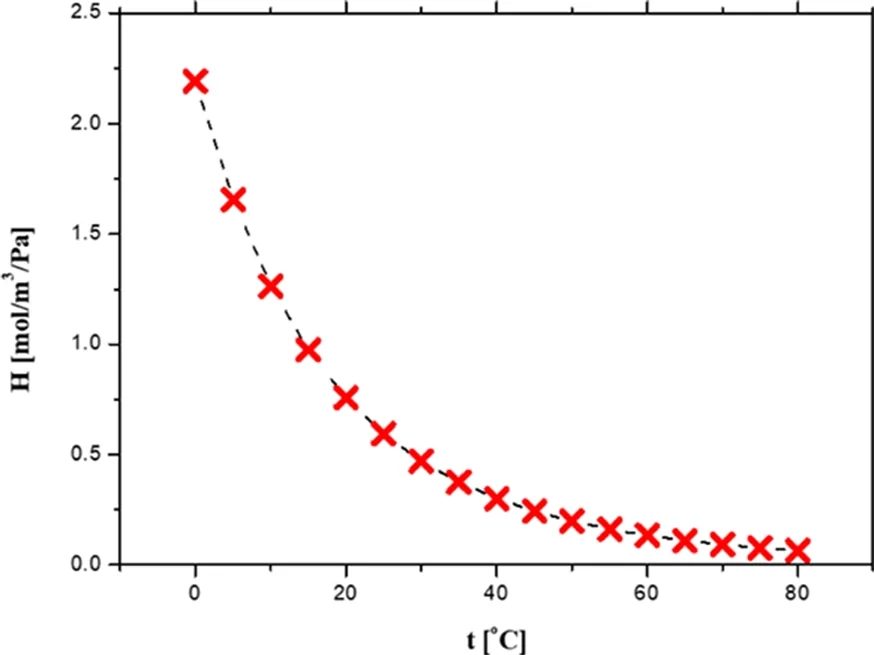
Temperature dependence of Henry’s constant

Equation 4 is a modified form of the van’t Hoff equation, and the values 4092 and 9.7 are constants derived from standard conditions and the enthalpy of dissolution. Knowing the standard Henry’s constant for ammonia (0.594 mol m$^{-3}$ Pa$^{-1}$) and the enthalpy of dissolution, it is therefore possible to calculate the dependence of Henry’s constant on temperature (see Fig. 2). Using this correlation, the theoretical amount of dissolved gas in water (or vice versa) can be calculated from the partial pressure of ammonia. Of course, this applies only under the assumption of ideal gas behavior and an infinitely diluted liquid solution.

Another key parameter influencing the absorption of ammonia into aqueous solution is the pH. Determination of the ratio between the ionic and molecular forms of ammonia requires knowledge of the dissociation constant $pK_{\textrm{b}}$, which at 25 $^\circ$C has a value of $pK_{\textrm{b}}$ = 4.74 (Vohlídal et al., 1999). The dissociation constant corresponds to the pH at which NH$_4^+$ and NH$_3$ are present in equal proportions. Calculations of the distribution between these two species are based on chemical equilibrium principles, even when the dissociation constant is affected by temperature. An empirical correlation describing the temperature dependence of the dissociation constant was proposed, for example, by Pitter (2009):

5 $$\begin{aligned} -\log K_{\mathrm {bH^+}} = 0.09018 + 2730\,T^{-1} \end{aligned}$$

**Fig. 3:**
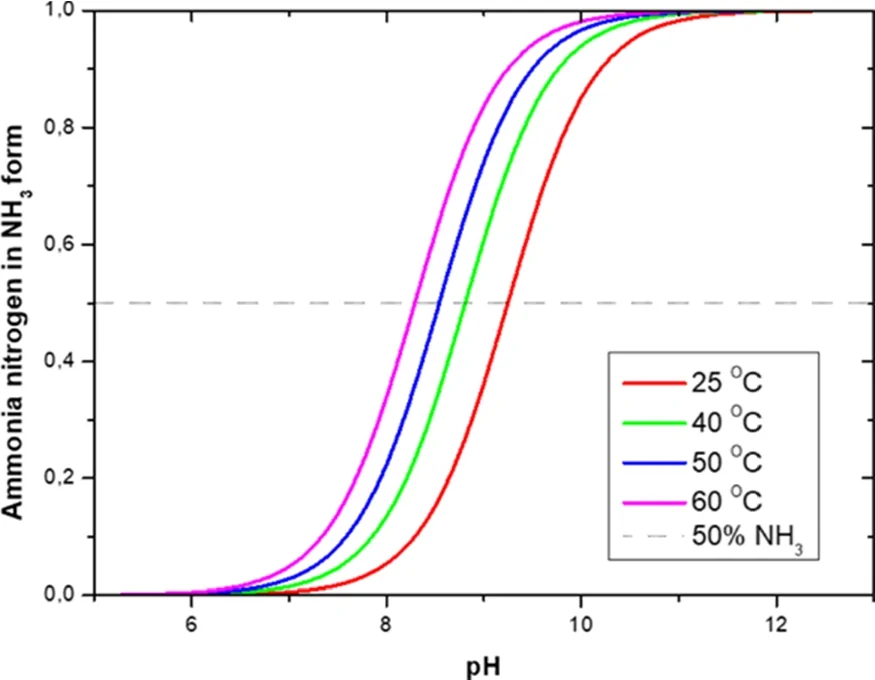
Effect of pH and temperature on ammonia NH$_3$ speciation

The distribution of ammonia at four different temperatures is illustrated in Fig. 3. It is evident that increasing temperature decreases the value of the dissociation constant. Consequently, a rise in temperature promotes the desorption of ammonia into its gaseous form.

**Table 2:** Mass transfer coefficients $K_\textrm{ov}$ for ammonia separation from various studies

Method	Membrane	pH$_{\textrm{feed}}$	*T*	Feed	Absorbent	$K_\textrm{ov}$	Eff.	Ref.
	material		($^\circ$C)			($\mathrm {m\,s^{-1}}$)	(%)	
MC	PP	10.0	–	Simulated wastewater	H$_2$SO$_4$	$9.2 \times 10^{-7}$	100	Serra-Toro et al. (2022)
MC	PP	10.2	35	Digestate	Acid solution	$2.6 \times 10^{-6}$	–	Shi et al. (2022)
MC	PTFE	10.2	35	Digestate	Acid solution	$7.3 \times 10^{-6}$	–	Shi et al. (2022)
MC	PVDF	11.0	25	Simulated wastewater	H$_2$SO$_4$	$1.5 \times 10^{-5}$	–	Tan et al. (2006)
MC	PP	13.0	20	Simulated wastewater	H$_2$SO$_4$	$1.4 \times 10^{-5}$	–	Zhu et al. (2005)
MC	PP	13.0	20	Simulated wastewater	H$_2$SO$_4$	$1.5 \times 10^{-5}$	–	Ashrafizadeh and Khorasani (2010)
MD	PP	9.0	45–75	Biogas slurry	Vacuum	0.3–$1.5 \times 10^{-6}$	–	He et al. (2018)
MD	PVDF	12.0	–	Simulated wastewater	Vacuum	$5.57 \times 10^{-5}$	99.5	Qu et al. (2013)
MD	PVDF	11.0	49	Landfill leachate	Vacuum	–	62	Zico et al. (2021)

**Abbreviations:** *MC* membrane contactor, *MD* *membrane distillation*, *PP* *polypropylene*, *PTFE* polytetrafluoroethylene, *PVDF* polyvinylidene fluoride

### New technologies in ammonia separation

For example, the separation of ammonia from wastewater produced by acidogenic fermentation using a membrane contactor was investigated by Serra-Toro et al. (2022). In their experiments, they examined the effect of elevated pH and temperature on both model and real ammonia-nitrogen solutions. For contacting the wastewater with the acidic absorbent solution (H$_2$SO$_4$), they employed hollow-fiber membrane contactors with an interfacial area of 0.5 m$^2$. In addition, the authors used a peristaltic pump for dosing acid into the absorbent solution, which maintained the pH of the solution at 1.0. The results show that, for the model solutions, mass transfer was most efficient at pH 10, taking into account the chemical consumption. Under these conditions, the mass transfer coefficient reached a value of $9.2 \times 10^{-7}$ m s$^{-1}$. In experiments with biowaste, the obtained coefficient was $5.0 \times 10^{-7}$ m s$^{-1}$. The authors stated that the lower mass transfer coefficient in the real sample was due to the simultaneous transfer of other interfering substances in addition to ammonia. An interesting comparison can be made with the study by Shi et al. (2022), who also investigated ammonia transfer from biogas plant digestate through membrane contactors. However, before entering the membrane module, the digestate was connected to an air pump. The authors used air bubbling inside the membrane module to promote more effective desorption (stripping) and subsequent transfer of ammonia into an NH$_4$H$_2$PO$_4$ absorbent solution. In their experiments, the digestate temperature was maintained at 60–70 $^\circ$C, while the absorbent was cooled to 30 $^\circ$C. Due to this temperature difference, membrane distillation occurred, and water vapor also permeated through the membrane pores. This observation is consistent with other studies on membrane distillation, which show that unequal temperature or pressure conditions across the membrane lead to water vapor transfer. The comparison between membrane distillation and membrane contactor separation parameters is shown in Table 2. The overall mass transfer coefficient for ammonia in experiments without aeration and pH adjustment reached $1.21 \times 10^{-7}$ m s$^{-1}$. The best results were achieved at a flow rate of 0.6 L min$^{-1}$, where the coefficient reached $4.85 \times 10^{-7}$ m s$^{-1}$. This value corresponds well to the results obtained by Serra-Toro et al. (2022), although it should be noted that Shi et al. (2022) conducted their experiments with an interfacial contact area of only 0.165 m$^2$. The study demonstrated that aeration of the digestate increases the mass transfer efficiency in membrane contactors. As the air flow rate increased from 0 to 0.6 L. $\text {min}^{-1}$, the mass transfer coefficient also increased. For instance, Ashrafizadeh and Khorasani (2010) increased the ammonia solution pH up to 13, achieving a mass transfer coefficient of $1.46 \times 10^{-5}$m s$^{-1}$. However, this approach is not economically viable and can be used only to accelerate the study of mass transfer kinetics.

An alternative to membrane-based processes is ammonia desorption using a carrier gas stream, i.e., the stripping process. An experimental study on ammonia removal from municipal wastewater by air stripping was conducted by Zangeneh et al. (2021). The authors evaluated the stripping efficiency at different pH values (9.70, 10.93, and 11.94), temperatures (34.25, 38.57, and 40.50 $^\circ$C), initial ammonia-nitrogen concentrations (27–98 mg L$^{-1}$), and liquid-to-gas flow ratios (60:1, 70:1, and 80:1). The experiments were carried out in a packed column made of plexiglass, with a height of 1 m, an inner diameter of 10 cm, and a 0.6 m layer of plastic packing particles (25 × 10 mm) to increase the interfacial area. According to the results, the maximum ammonia removal efficiency was 98%, achieved at the highest tested pH and temperature (pH 11.94 and 40.5 $^\circ$C) and at a liquid-to-gas ratio of 80:1, consistent with theoretical expectations. This is consistent with other studies, which used elevated temperatures (40–60 $^\circ$C) or increased pH for ammonia stripping, achieving efficiencies between 89% and 100% (Değermenci et al., 2012; Marttinen et al., 2002; Zhu et al., 2017).

Another interesting method for ammonia removal was investigated by Zhang et al. (2019), who studied the adsorption of ammonia onto amorphous sodium titanate nanoparticles. The model wastewater contained 20 mg L$^{-1}$ of ammonium nitrogen. The study included the synthesis and morphological characterization of the adsorbent and examined the effects of temperature, pH, initial adsorbent concentration, and the presence of other ions on adsorption efficiency. The authors found that the maximum adsorption capacity was achieved at an adsorbent concentration of 2 g L$^{-1}$, which was therefore used in all subsequent experiments. For TiO$_2$ and sodium titanate, adsorption rate increased with pH up to pH 4, remained constant until pH 9, and then decreased. In contrast, pH had little influence on TiO$_2$, and its adsorption efficiency was lower than 20%. On the other hand, sodium titanate had an adsorption efficiency near 75%. High concentrations of cations (Na$^+$, K$^+$, Ca$_2^{\,\,+}$, Mg$_2^{\,\,+}$) slightly reduced adsorption, though this effect is negligible in real wastewaters. The data fitted best to the Langmuir isotherm, indicating monolayer adsorption on a homogeneous surface, with a maximum adsorption capacity of 44.54 mg g$^{-1}$ at 25 $^\circ$C.

In addition to conventional physicochemical processes, recent advances have expanded the understanding of microbial pathways for pollutant separation from wastewater. This process has been investigated through various microbial groups, including ammonia-oxidizing bacteria (AOB) and ammonia-oxidizing archaea (AOA). AOA can be divided into soil-associated groups, represented by *Nitrososphaera viennensis*, and marine groups, such as *Nitrosopumilus maritimus*. All cultivated AOA are considered chemolithoautotrophs, as they derive energy from ammonia oxidation and fix carbon dioxide into biomass. In contrast, AOB are well-studied microorganisms that catalyze the oxidation of ammonia and belong to the class *Gammaproteobacteria*. These microorganisms can utilize reduced inorganic nitrogen species as substrates. The overall nitrification process consists of two steps: in the first step, ammonia is oxidized to hydroxylamine, catalyzed by the enzyme ammonia monooxygenase, which is embedded in the microbial membrane (Eq. C.1). In the second step, hydroxylamine is oxidized to nitrite in the presence of the enzyme hydroxylamine oxidoreductase (Eq. C.2).

C.1 $$\begin{aligned} \text {NH}_3 + \text {O}_2 + \text {2H}^+ + 2\text {e}^- \longrightarrow \text {NH}_{2}\text {OH} + \text {H}_{2}\text {O} \end{aligned}$$

C.2 $$\begin{aligned} \text {NH}_{2}\text {OH} + \text {H}_{2}\text {O} \longrightarrow \text {NO}_{2}^{\,\,-} + \text {5H}^{+} + 4\text {e}^{-} \end{aligned}$$

This entire process serves as a rich source of energy for both AOA and AOB, supporting their growth and survival. However, a major drawback of these processes is that the activity of nitrifying bacteria leads to the formation of greenhouse gases mainly in the denitrification process, where NO$_2^{\,\,-}$ is reduced to nitrous oxide and N$_2$ through the reactions described by Eqs. C.3–C.5:

C.3 $$\begin{aligned} \text {NO}_2^{\,\,-} + \text {2H}^+ + \text {e}^- \longrightarrow \text {NO} + \text {H}_2\text {O} \end{aligned}$$

C.4 $$\begin{aligned} \text {2NO} + \text {2H}^+ + \text {2e}^- \longrightarrow \text {N}_2\text {O} + \text {H}_2\text {O} \end{aligned}$$

C.5 $$\begin{aligned} \text {N}_2\text {O} + \text {2H}^+ + \text {2e}^- \longrightarrow \text {N}_2 + \text {H}_2\text {O} \end{aligned}$$

However, not all $\text {N}_2\text {O}$ is reduced, and a fraction of the produced gas can escape to the atmosphere and contribute to global warming. Overall, $\text {N}_2\text {O}$ accounts for approximately 6% of global warming, while the proportion specifically attributable to nitrification processes is difficult to quantify, as it strongly depends on climatic conditions, soil type, and local environmental factors (Inatomi et al., 2019; Zhu et al., 2025). Moreover, under real environmental conditions, opposing processes such as ammonification may occur simultaneously, during which ammonia is produced by bacteria (Al-Ajeel et al., 2026; Soliman & Eldyasti, 2018).

Partial nitritation–anammox (PN/A) has emerged as one of the most promising biological alternatives to conventional nitrification–denitrification, particularly for the treatment of ammonium-rich wastewaters with low biodegradable organic carbon content. In this shortcut pathway, approximately half of the ammonium is aerobically oxidized to nitrite, while the remaining ammonium is directly converted together with nitrite into nitrogen gas by anaerobic ammonium-oxidizing (anammox) bacteria. Compared with conventional nitrification–denitrification, PN/A substantially reduces aeration demand, eliminates the need for external organic carbon, and decreases excess sludge production, resulting in lower operational costs and improved energy efficiency. Depending on wastewater characteristics and reactor configuration, reported nitrogen removal efficiencies generally range from 70 to over 90%, while optimized systems have achieved ammonia removal efficiencies approaching 98–99% (Ali & Okabe, 2015).

PN/A performs best in ammonium-rich wastewaters, typically containing around 1500 mg NH$_4^{\,\,+}$ l$^{-1}$, and under conditions that allow stable partial nitritation. Successful operation requires strict control of dissolved oxygen, since excessive oxygen inhibits anammox bacteria whereas insufficient oxygen limits ammonia oxidation. Another critical operational parameter is nitrite concentration, as nitrite serves as an essential substrate but becomes inhibitory at elevated concentrations; levels above approximately 400 mg $\text {NO}_2^{\,\,-}$ l$^{-1}$ have been reported to be toxic to anammox microorganisms. In addition, lower operating temperatures significantly reduce anammox growth rates, making stable mainstream applications considerably more challenging (Ali & Okabe, 2015; Guo et al., 2025).

Since the commissioning of the first full-scale PN/A plant, which achieved a nitrogen loading rate of 7.1 kg N ${\text {m}^{-3}\,\text {d}^{-1}}$ after 1235 days of operation, the technology has evolved into one of the most mature shortcut biological nitrogen removal processes. Initially developed for sidestream treatment of high-strength wastewaters, PN/A has since been successfully implemented in numerous full-scale wastewater treatment plants, particularly in Europe. Current research focuses on extending its application to mainstream municipal wastewater treatment through improved reactor configurations, granular sludge and biofilm systems, advanced process control, and integration with nutrient recovery processes (Guo et al., 2025).

Simultaneous nitrification–denitrification (SND) represents another promising biological strategy for nitrogen removal, enabling both nitrification and denitrification to occur simultaneously within a single reactor. Unlike conventional biological nitrogen removal, which requires separate aerobic and anoxic zones, SND relies on oxygen gradients naturally formed inside activated sludge flocs, biofilms, or granular sludge. While aerobic microorganisms oxidize ammonium near the biomass surface, oxygen-limited microenvironments within the biomass support denitrification, allowing nitrogen oxides produced during nitrification to be directly converted into nitrogen gas. Consequently, SND simplifies reactor configuration, reduces reactor volume, and can decrease both aeration requirements and the demand for external carbon sources (James & Vijayanandan, 2023).

The efficiency of SND strongly depends on maintaining a balance between nitrification and denitrification. Dissolved oxygen concentration is considered the most critical operational parameter, as excessive aeration suppresses anoxic microzones required for denitrification, whereas insufficient oxygen limits nitrification. Other important factors include the carbon-to-nitrogen ratio, sludge retention time, biomass structure, and reactor configuration, all of which influence oxygen diffusion and microbial community composition. Consequently, stable SND performance requires careful process control to maintain favorable conditions for both microbial groups simultaneously (Loh et al., 2023).

Simultaneous nitrification–denitrification has been successfully implemented in a variety of municipal and industrial wastewater treatment plants, demonstrating its potential as a practical alternative to conventional biological nitrogen removal. Depending on wastewater characteristics and reactor configuration, total nitrogen removal efficiencies commonly exceed 80%, while optimized biofilm systems have reported efficiencies approaching 98%. Despite these promising results, stable full-scale performance remains highly dependent on reactor design and operational control, particularly with respect to dissolved oxygen concentration and the maintenance of aerobic and anoxic microenvironments. Consequently, current research focuses on improving process stability through advanced biofilm carriers, granular sludge technologies, optimized aeration strategies, and integrated process control to facilitate broader full-scale implementation (González-Tineo et al., 2022).

**Table 3:** Comparative overview of the principal ammonia treatment and recovery technologies synthesized from the literature reviewed in this article. Representative reviews supporting each technology are provided in the last column

Technology	Typical application	Key advantage	Key limitation	Status	Representative review
Conventional nitrification–denitrification	Municipal wastewater	Robust and reliable process	High aeration and external carbon demand	Widely applied	Farghali et al. (2024)
Partial nitritation–anammox (PN/A)	High-strength sidestreams	Low aeration demand; no external carbon requirement	Strict dissolved oxygen and nitrite control	Commercially available	Ali and Okabe (2015); Guo et al. (2025)
Simultaneous nitrification–denitrification (SND)	Municipal and industrial wastewater	Compact single-reactor process	Maintenance of aerobic and anoxic microzones	Commercially available	James and Vijayanandan (2023); Loh et al. (2023)
Feammox	Anaerobic ammonium-rich wastewater	Anaerobic nitrogen removal without aeration	Slow kinetics and limited practical experience	Research stage	Wan et al. (2022); Xia et al. (2022)
Air stripping	Digestate, landfill leachate, and industrial wastewater	Simple and effective ammonia recovery	High alkali and acid consumption	Widely applied	Farghali et al. (2024)
Membrane contactor	Ammonia recovery and fertilizer production	High recovery efficiency and product quality	Membrane wetting and fouling	Commercially available	Darestani et al. (2017)
Struvite precipitation	Nutrient-rich wastewater	Simultaneous nitrogen and phosphorus recovery	Magnesium demand and scaling	Commercially available	Farghali et al. (2024)
Ion exchange	Municipal and industrial wastewater	High selectivity for ammonium removal	Resin regeneration and regenerant management	Established process	Zhou et al. (2024); Farghali et al. (2024)
Adsorption	Municipal and industrial wastewater	Simple operation and broad adsorbent selection	Limited capacity and adsorbent regeneration	Established process	Zhou et al. (2024); Farghali et al. (2024)
Electrochemical technologies	Ammonia-rich wastewater	Compact and potentially chemical-free operation	High electricity demand and electrode stability	Emerging	Farghali et al. (2024); Chen and Kim (2025)

*Status definitions:* *Widely applied* indicates technologies routinely implemented in municipal and/or industrial wastewater treatment. *Commercially available* indicates that dedicated commercial systems based on the respective technology are currently available on the market. Representative examples include ANAMMOX^®^ (Paques) for partial nitritation–anammox (PN/A), OxyMem MABR systems for simultaneous nitrification–denitrification (SND), Liqui-Cel^™^ membrane contactors (Solventum) for membrane-based ammonia recovery, and the Pearl^®^ process (Ostara) for struvite recovery. *Established process* refers to mature technologies that are widely used in water and wastewater treatment but are generally implemented using various commercially available materials or equipment rather than a single dedicated technology platform. *Emerging* refers to technologies with demonstrated practical potential but still limited large-scale implementation, whereas *Research stage* denotes technologies that remain predominantly under laboratory or experimental investigation

Ferric iron-mediated anaerobic ammonium oxidation (Feammox) is another emerging biological pathway for ammonium removal, first described by Clement et al. (2005), in which ammonium is oxidized under anoxic conditions using ferric iron Fe(III) as the terminal electron acceptor instead of oxygen. Unlike conventional nitrification, the process proceeds without aeration and may produce nitrogen gas, nitrite, or nitrate depending on environmental conditions and the composition of the microbial community. This pathway established a previously unrecognized link between the nitrogen and iron biogeochemical cycles and has attracted increasing attention as a potential low-energy alternative for nitrogen removal.

Compared with conventional biological nitrogen removal, Feammox eliminates the need for oxygen supply and external organic carbon, making it particularly attractive for wastewaters with low C/N ratios and oxygen-limited environments. However, its practical application remains limited due to relatively low nitrogen conversion rates, incomplete understanding of the underlying microbial mechanisms, and the need to maintain sufficient bioavailable Fe(III) through efficient iron regeneration. Current research is therefore focused on identifying additional Feammox microorganisms, clarifying metabolic pathways, improving Fe(II)/Fe(III) cycling, and integrating Feammox with complementary biological processes such as anammox to enhance overall nitrogen removal efficiency and facilitate future full-scale applications (Wan et al., 2022; Xia et al., 2022).

As summarized in Table 3, no single technology simultaneously maximizes ammonia removal efficiency, nutrient recovery, operational simplicity, and economic feasibility. Instead, each technology occupies a specific application niche determined by wastewater characteristics, treatment objectives, and operational constraints. Conventional biological processes remain the dominant solution for municipal wastewater treatment because of their robustness and widespread implementation, whereas recovery-oriented technologies, such as membrane contactors, air stripping, and struvite precipitation, are particularly advantageous for concentrated ammonia streams where nutrient recovery is economically justified. Emerging biological pathways, including partial nitritation–anammox (PN/A) and Feammox, offer significant potential to reduce energy demand and improve process sustainability; however, their broader implementation remains constrained by process stability and operational complexity. Therefore, technology selection should be based on an integrated evaluation of wastewater composition, recovery objectives, operational requirements, environmental impacts, and economic feasibility rather than on ammonia removal efficiency alone.

### Smart fertilizers

An important focus of current research is the control of the timing and magnitude of ammonia release, which represents a conceptually opposite strategy to the separation and treatment of ammonia after it has already entered the environment. However, a strong synergy exists between these two approaches, as the appropriate design of coated and smart fertilizers can significantly suppress short-term concentration peaks that otherwise dominate environmental impacts. Controlled-release and coated fertilizers (e.g., polymer- or sulfur-coated urea) are designed to delay nitrogen availability and smooth ammonia release. Field studies typically report reductions in NH$_3$ volatilization in the range of 8–37%, depending on coating type and environmental conditions such as soil moisture and temperature (Cancellier et al., 2016; Shan et al., 2015; Shaviv, 2001). Among enhanced-efficiency fertilizers, urease inhibitors reduced NH$_3$ volatilization by approximately 54%, while controlled-release fertilizers achieved reductions of about 68%. Nitrification inhibitors modify nitrogen transformation pathways and mobility, indirectly affecting ammonium persistence, although their effectiveness varies and potential trade-offs, including impacts on other nitrogen emissions, must be carefully considered (Akiyama et al., 2010; Pan et al., 2016). Importantly, these upstream measures alter the temporal distribution of ammonia release, leading to lower peak concentrations but more prolonged low-level emissions. From a technological perspective, this shift affects both monitoring requirements—favoring high-resolution or time-integrated approaches—and the design and operation of separation and recovery systems, which must function efficiently under more variable and often lower influent concentrations. Overall, effective ammonia management increasingly relies on integrated strategies that combine upstream emission control with downstream monitoring, separation, and recovery at controllable hotspots, supported by data-driven process optimization.

## Utilization of recovered ammonia

### Ammonia salts

Ammonia readily forms salts with both acids and bases. However, the ammonium bond is more stable in acidic environments, whereas in alkaline conditions, part of the TAN may volatilize as gaseous NH$_3$. A key compound is ammonium chloride, widely used as a fertilizer component and in industry as an electrolyte in dry cells or a hardener in formaldehyde adhesives. Its production, together with sodium bicarbonate, occurs in the Solvay process, where the two compounds are subsequently separated, purified, and dried (Othmer & Kirk, 2007; Steinhauser, 2008). An example illustrating the importance of this process is the study by Sudibyo et al. (2022), who investigated the operating conditions of the Solvay process and performed statistical evaluation using the Taguchi method. The key parameters influencing the formation of sodium bicarbonate were the reaction time, initial NaCl concentration, and the $\text {CO}_2$ flow rate. Typically, the Solvay process employs a column with two gas inlets ($\text {CO}_2$ and $\text {NH}_3$); however, in this study, a 20% $\text {NH}_4\text {OH}$ solution was used as a source of both ammonia and water. This substitution was chosen for economic reasons, as aqueous ammonia is more cost-effective than gaseous ammonia. The chemical reaction proceeded according to Eq. C.6, with ammonia consumption determined stoichiometrically.

C.6 $$\begin{aligned} \text {NH}_4\text {OH} + \text {CO}_2 + \text {NaCl} \longrightarrow \text {NaHCO}_3 + \text {NH}_4\text {Cl} \end{aligned}$$

Other important ammonium compounds include ammonium nitrate, ammonium sulfate, and ammonium phosphate, which are primarily used as fertilizers. Ammonium nitrate also serves as a precursor in explosive production and, when heated above 200 $^\circ$C, for the generation of nitrous oxide for medical use. The simplest method for preparing these compounds is the absorption of ammonia into the corresponding inorganic acids (H$_2$SO$_4$ or $\text {HNO}_3$), similarly to the synthesis of ammonium chloride, although other acids can also be employed. Both inorganic acids ($\text {HNO}_3$, H$_2$SO$_4$, $\text {H}_3\text {PO}_4$) and organic acids have been explored as absorbents for ammonia recovery (Aligwe et al., 2020; Cifuentes-Cabezas et al., 2023; Ge et al., 2023). Among these, sulfuric acid (H$_2$SO$_4$) and phosphoric acid ($\text {H}_3\text {PO}_4$) demonstrate the best overall performance. At the beginning of operation, $\text {H}_3\text {PO}_4$ often provides a higher initial ammonia removal rate due to its rapid proton exchange and neutralization reaction with NH$_3$. However, during prolonged operation, its efficiency declines faster than that of H$_2$SO$_4$, mainly because of ammonium phosphate precipitation and mass-transfer limitation within the membrane. In contrast, H$_2$SO$_4$ maintains greater long-term stability and absorption capacity, making it the preferred and more cost-effective option for continuous ammonia recovery (Reig et al., 2021). Although organic acids (e.g., di(2-ethylhexyl) phosphoric acid, $\text {CH}3\text {COOH}$) and alternative absorbents (e.g. $\text {NaHCO}_3$, spent pickling brine) have been tested, their mass-transfer coefficients are generally lower, and their reactions proceed more slowly, restricting their industrial application (Li et al., 2024).

### Struvite precipitation

Separated ammonia can also be used to precipitate struvite (magnesium ammonium phosphate hexahydrate), which is also an effective agricultural fertilizer (Latifian et al., 2012). This is a simple process in which gaseous ammonia bubbles through a solution containing magnesium and phosphate ions. However, it is important to observe several conditions to ensure nucleation and subsequent crystal growth. The main parameters affecting struvite precipitation are the ion ratios in the initial solution and the pH value, with temperature being less important (Wang et al., 2005). Intensive laboratory research is currently underway in the field of struvite precipitation for the simultaneous capture of TAN and phosphates from wastewater. Nelson et al. (2003) report in their work that the lowest solubility of struvite, and thus the most effective precipitation, occurs at a pH of 8.90–9.25. They also report that when studying the effect of the Mg:P molar ratio, the best results were achieved with a combination of 1.2:1.0. The group of González-Morales et al. (2021) also studied the effect of temperature on the precipitation and purity of struvite from digestate, in addition to the effect of pH. In the case of pH, the most effective precipitation occurred at pH 9, and the same molar ratio of nitrogen, phosphorus, and magnesium, and the ideal temperature was determined to be 25 $^\circ$C according to the experiment. At higher temperatures, part of the ammonia evaporated, and therefore, the authors recommend placing a column with an acidic absorption solution behind the precipitation reactor. Compliance with these recommended conditions minimizes the precipitation of other impurities, and it has also been confirmed that high concentrations of TAN in the reaction mixture increase the purity of the precipitated struvite. Stratful et al. (2001) report more than 97% transformation of magnesium ions into struvite at a pH range of 9–10 using equimolar amounts of Mg, P, and N. Among other things, it has also been confirmed that an excess of ammonia is very beneficial for struvite precipitation. For a reaction lasting 60 min, the length of the resulting crystals was found to be 0.8 mm, and when the reaction time was extended to 180 min, their length increased to 3 mm.

For effective precipitation of struvite from wastewater and liquids, it is necessary to supply magnesium ions to the system and, in the case of waste with a higher TAN content, also phosphates. Magnesium chloride and magnesium sulfate are ideal sources of magnesium, but increasing the chloride and sulfate content in the solution reduces the quality of the product. Another alternative is magnesium oxide, but its use has disadvantages. Magnesium oxide is poorly soluble in water, and to ensure a sufficient amount of magnesium ions, its amount must be increased by more than 1.5 times, as reported in a study by Quintana et al. (2004). There are other forms of magnesium that have been tested for struvite formation, such as metallic magnesium with the need for reduction, magnesium carbonate, and seawater (Huang et al., 2015; Rubio-Rincón et al., 2014). Magnesium carbonate was used as a source of magnesium in the work of Gunay et al. (2008). They solved the low solubility of magnesite by adding hydrochloric acid. The stoichiometric course of the reaction resulted in the formation of magnesium chloride, in which form the magnesium dissociated and was transformed into struvite. Unfortunately, the authors do not mention how they dealt with the resulting carbon dioxide, which was released into the air from the stable form of carbonate. It is important to note that regardless of the source of magnesium ions, the efficiency of ammonia removal is similar and in all cases exceeds 90%. As already mentioned, phosphorus must also be added to liquids containing excess TAN. Phosphoric acid and its sodium and potassium salts have proven to be very effective compounds for struvite formation (Kabdaşlı & Tünay, 2018; Siciliano et al., 2020).

In general, an equimolar amount of nitrogen, phosphorus, and magnesium is ideal for struvite precipitation. The Mg:P ratio is crucial, which is reported in the literature as 1:1, or with a small excess of magnesium ions depending on the source of magnesium. In addition, it has been confirmed that the solubility of struvite increases with increasing temperature, so it is important to work at lower concentrations.

### Ammonia for carbon dioxide capture

Ammonia and its compounds are important absorbers of carbon dioxide. Given the increasingly stringent restrictions on emissions due to climate change and global warming, this property of ammonia is advantageous, and this topic is increasingly the subject of studies. One possible method is the absorption of $\text {CO}_2$ into an aqueous ammonia solution to form ammonium bicarbonate and ammonium carbonate. Another method is the absorption of carbon dioxide into solutions of organic amines derived from ammonia. These are mainly the simplest amines such as ethylamine, diethylamine, or diethanolamine. Amines and NH$_3$-based absorbents are widely used, but their use is limited by high regeneration energy, corrosiveness, evaporation of the reagent, and secondary pollution caused by NH$_3$ leakage (Ochedi et al., 2021).

The absorption of carbon dioxide into aqueous ammonia solutions is a highly effective separation method that reduces environmental impact. Its main drawbacks include the high volatility of ammonia, which results in higher energy requirements for its regeneration, and the precipitation of solids, which interferes with the carbon dioxide regeneration process (Yu & Wang, 2015). In a study by Liu et al. (2009), it was found that the initial efficiency of carbon dioxide removal by absorption into an aqueous ammonia solution can reach up to 90% when using an aqueous ammonia solution with an ammonia concentration of more than 5%. A direct correlation between the concentration of ammonia in water and the efficiency of carbon dioxide removal has been demonstrated; however, at ammonia concentrations higher than 15%, a large amount of ammonia evaporated. The authors mention that low ammonia concentrations may also have higher removal rates and therefore recommend aqueous ammonia concentrations of 5 to 10%. Hamouda et al. (2020) also confirmed that very low ammonia concentrations (1, 3, and 7%) are sufficient to achieve 70–85% efficiency. In the experimental part, they examined the effect of temperature on carbon dioxide absorption and found that the absorption rate decreases linearly with increasing temperature between 5–30 $^\circ$C. This is due to more intense evaporation of ammonia and lower production of ammonium bicarbonate. The time required to achieve maximum carbon dioxide removal efficiency (90%) was 30 min.

Although carbon capture is not a primary ammonia-removal technology, the use of recovered ammonia for $\text {CO}_2$ capture represents an additional valorization route and an example of cross-sectoral synergy within the circular economy. By linking nitrogen recovery from wastewater with carbon management, this approach illustrates how recovered ammonia can serve as a secondary feedstock beyond its conventional use in fertilizer production.

The reviewed recovery pathways demonstrate that the value of recovered ammonia strongly depends on product quality, local market demand, and integration with existing industrial processes. Consequently, successful resource recovery should be evaluated not only according to recovery efficiency but also considering product utilization, transportation requirements, and economic feasibility. This shift from waste treatment towards resource management represents one of the key principles of the circular economy.

## Chemical analysis methods

In order to determine the effectiveness of TAN removal from wastewater, it is necessary to optimize the analytical method used for its determination. The analytical method must be optimized according to the technology used and the composition of the TAN-containing matrix. One method of analysis is to monitor the accumulation of ammonia in an aqueous absorption solution. The second method is to analyze the initial solution and determine the ammonia loss, which can be more complicated in the case of a turbid, non-homogeneous suspension. One effective alternative is to measure the concentration of ammonia in the gas phase, but this is limited to technologies that use desorption of ammonia from liquid wastewater into gas form. The following chapter will discuss the effectiveness and applicability of methods for determining TAN and its individual forms. It is also important to mention that if the sample is not clear, ammonia distillation followed by absorption into an acid solution can be used (Li et al., 2020). This method converts and stabilizes ammonia into its ionic form in a clear solution, where it can be analyzed using all the methods presented below.

### Titration methods

Given the high volatility of ammonia and its complex behavior in aqueous environments, a back-titration method can be employed for its quantification. In this method, a known excess of hydrochloric acid (HCl) is added to the sample, converting ammoniacal nitrogen into ammonium chloride (NH$_4$Cl) under acidic conditions. The unreacted HCl is then determined by titration with a standard sodium hydroxide (NaOH) solution using phenolphthalein as an indicator. This procedure is particularly useful for screening-level determinations with lower sensitivity; accurate application requires that all standard solutions be regularly standardized (Liu et al., 2017). In addition to spectrophotometric procedures, ammonia can be quantified by potentiometric (pH-metric) titration of the absorbing solution, a workflow referenced in Standard Methods and commonly implemented in automated titration systems (Bridgewater et al., 2017).

### Spectral methods

**Fig. 4:**
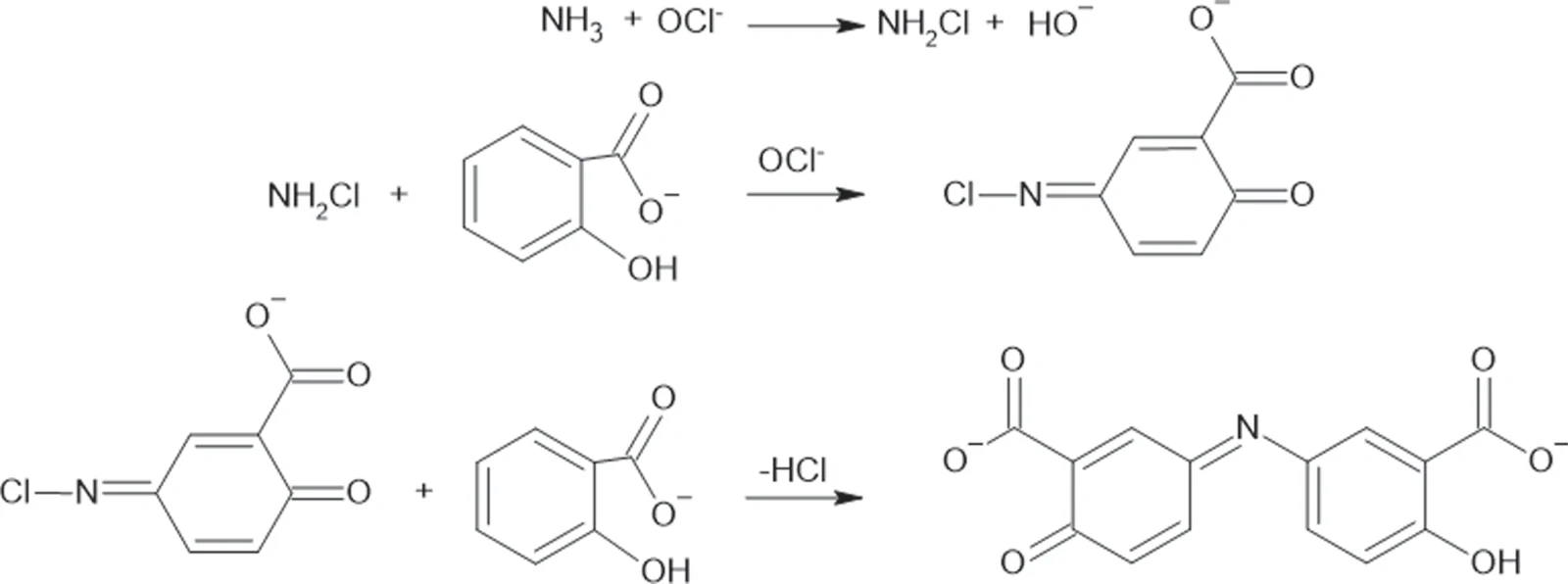
Determination of ammonia by the indophenol method

One of the best-known methods for determining ammonia in a clear solution is the Berthelot or indophenol method (see Fig. 4) (Cogan et al., 2014). The basic condition for performing this method is alkalization of the solution and conversion of ammoniacal nitrogen into molecular NH$_3$ form. The released ammonia is then oxidized to chloramine (in the presence of hypochlorite ions), which subsequently reacts with phenol or sodium salicylate to form a blue indophenol compound, which, however, in the presence of a sodium nitroprusside catalyst (yellow color), acquires the resulting green color. The intensity of the color is then directly proportional to the concentration of ammoniacal nitrogen in the sample and can be measured spectrophotometrically. When performing this analysis, it is necessary to maintain a constant temperature and prevent the occurrence of interfering ions in the sample. This method is described in the Czech Republic by the ČSN ISO 7150-1 standard (Český normalizační institut, 1994).

An alternative is the Nessler method (Eq. C.7), which uses mercuric iodide and potassium iodide in an alkaline solution as reagents. In the presence of ammonia in the solution, a yellow-colored compound is formed (Lin et al., 2019; Li et al., 2020). In the Nessler method, ammonia is quantified spectrophotometrically at  420  nm based on the formation of a mercury–amido complex, whereas the Berthelot (phenate) method relies on the formation of indophenol blue with a characteristic absorption maximum at  630–640 nm and a molar absorption coefficient on the order of ${1 \times 10^4}$ l mol$^{-1}$ cm$^{-1}$.

C.7 $$\begin{aligned} 2[\text {HgI}_{4}]^{2-} + \text {NH}_3 + 3\text {OH}^- \longrightarrow \text {HgO}\cdot \text {Hg}(\text {NH}_{2})\text {I} + \text {7I}^- + \text {2H}_2\text {O} \end{aligned}$$

Another option is determination by fluorescence using o-phthalaldehyde, which reacts with amino acids/ammonia in the presence of a reducing agent (Roth, 1971). Initially, 2-mercaptoethanol was used as a reducing agent, but later, Genfa and Dasgupta (1989) found that the use of sodium sulfite increases the selectivity of the method in favor of ammonia. The resulting compound is then able to emit fluorescent radiation at a specific wavelength and intensity that is directly proportional to the concentration of ammonia in the sample. This method is more sensitive than the spectrophotometric methods mentioned above, and fluorescence can be measured as early as 5 min after the mixture of reagents is formed. In addition, new fluorescence methods are currently being developed to improve the accuracy and sensitivity of ammonia analysis in natural samples, as summarized in the work of Li et al. (2020). Beyond the classical o-phthalaldehyde (OPA) method, several alternative fluorescent reagents and materials have been developed for ammonia detection, including methoxy-substituted phthalaldehydes (e.g., MOPA and M2OPA), which offer faster reaction kinetics and improved sensitivity down to the nanomoles range. Other approaches employ organic fluorescent dyes and functional materials, such as rhodamine, coumarin, oxazine derivatives, Schiff-base fluorophores, and quantum dots, benefiting from high photostability, strong emission, and tunable response. In addition, advanced fluorescence sensing platforms, including aza-BODIPY dyes, functional films, and integrated flow-based or microfluidic fluorescence systems, enable ultra-trace ammonium detection in complex environmental matrices and support miniaturized or in situ monitoring applications, as summarized in the work of Li et al. (2020).

### Electrochemical methods

Ammonia detection electrodes use a hydrophilic permeable membrane to separate ammonia from the aqueous sample with an internal ammonium chloride solution. When the pH of the solution is adjusted to above 11, the ammonium salt is converted to ammonia, which is then introduced into the internal liquid through the membrane, which can only allow ammonia to pass through. When the partial pressures of ammonia on both sides are equivalent, a potential difference arises, which means that the pH of the internal liquid has increased. This results in a change in pH on the glass electrode, and the potential signal is measured using a pH meter. A gas-sensing ammonia electrode is not a suitable option for determining ammonia in solution. The ammonia-permeable membrane is hydrophobic and does not allow water molecules or hydrated molecules to pass through. A problem arises if the sample is not an aqueous solution and does not wet the membrane well, preventing the diffusion of liquid. This can also be caused by the presence of surfactants in the sample. Another disadvantage is the lifespan of the porous membrane, which must be replaced every 2 to 3 weeks (Freiser, 1978).

In contrast, ion-selective electrodes (ISEs) for ammonium ions exhibit high ion selectivity and sensitivity, which supports a short response time. Therefore, it is advantageous to use these electrodes both for routine solution checks and for monitoring the concentration of relevant ions in operation. The basis of an ion-selective electrode is a membrane capable of selectively reacting to ammonium ions. The most commonly used membrane for ammonium ions is based on polyvinyl chloride. When this ISE is placed in a liquid sample, a potential is created between the inside and outside of the sensitive membrane. By combining the measured potential and the Nernst equation, the concentration of ammonium ions in the sample can be calculated (Wen et al., 2019).

### Other advanced methods

For example, chromatographic techniques provide com-plementary tools for ammonia determination depending on the phase and analytical objective. Ion chromatography (often informally referred to as ion HPLC) is primarily used for the determination of ammonium (NH$_4^+$) in aqueous samples, where separation on an ion-exchange column enables selective and sensitive quantification, frequently together with other major inorganic ions. This makes the method suitable for reference analysis and validation in complex matrices, although at the expense of instrument cost and sample preparation requirements (Noller & Currey, 1990). In contrast, gas chromatography, with or without mass spectrometric detection, is mainly applied to gas-phase ammonia, for example in off-gas streams from stripping or membrane-based separation processes. Its use for aqueous ammonium typically requires additional conversion steps and is therefore limited to specialized applications rather than routine water analysis (Shiraki et al., 2023).

Flow analysis techniques, including flow injection analysis (FIA) and continuous flow analysis (CFA), are based on the controlled transport of a sample through a flow manifold, where reagents are added and the analytical reaction proceeds under reproducible hydrodynamic conditions. Detection is most commonly spectrometric. In typical configurations, ammonia is released from an alkalized sample and transferred across a gas-permeable membrane into a receiving stream. There, ammonia reacts with a suitable reagent, such as o-phthalaldehyde, forming a fluorescent product that is amplified by a photomultiplier and measured at excitation and emission wavelengths of approximately 365 nm and 430 nm, respectively (FIALab Instruments, 2018; Liang et al., 2016). An alternative approach employs a diffusion cell with a receiving solution containing a pH indicator, where the continuous change in color intensity is monitored as a function of ammonia concentration. These principles enable high-throughput and fully automated ammonium determination and can be readily adapted to at-line or online configurations, making flow analysis particularly suitable for routine monitoring and real-time process control in wastewater treatment plants and ammonia separation systems (Český normalizační institut, 2005).

Also, passive sampling techniques provide time-integrated information on labile ammonium fractions, offering an alternative to instantaneous measurements obtained by grab sampling or high-frequency sensors. In the diffusive gradients in thin films (DGT) approach, ammonium diffuses through a well-defined gel layer and is immobilized on a selective binding phase, enabling the determination of a time-averaged concentration. As a diffusion/binding layer for ammonia, materials such as the cation-exchange resin Microlite PrCH or zeolite-based binding gels can be used, with subsequent spectrophotometric detection based on the Nessler or indophenol method (Feng et al., 2015; Huang et al., 2016). This integration over time makes DGT particularly well suited for environmental applications in surface waters, sediments, and porewaters, where episodic concentration peaks and strong spatial heterogeneity are common and difficult to capture using conventional sampling strategies (Davison & Zhang, 1994). A comparative overview of the analytical methods is provided in Table 4.

**Table 4:** Analytical methods for ammonia determination

Method	LOD	Advantages	Disadvantages	Ref.
	(mg TAN L$$^{-1}$$)			
Distillation–titration	5	Robust for high TAN	Time-consuming	Bridgewater et al. (2017)
Nessler	0.01	Simple; sensitive colorimetry	Uses mercury reagent	ASTM International (2021)
Berthelot	0.02	Widely standardized	Color interferences	ASTM International (2021)
Fluorescence	0.0001	Low LOD; adjustable	Lab-based	Li et al. (2020)
ISE	0.0042	Portable; low cost	Interference (K$^{+}$)	Gil et al. (2022); Thermo Fisher Scientific (2026)
IC, HPLC	0.00153	High selectivity	Instrument cost	Bragulla et al. (2026)
GC	0.00011	Excellent for gas	Not for aqueous NH$_4^+$	Zaffaroni et al. (2020)
Flow Analysis	0.1	Standardized; robust routine monitoring	Reagents; maintenance	Český normalizační institut (2005)
DGT	0.00063	Captures cumulative exposure	No instantaneous values; deployment and post-analysis	Huang et al. (2016); Kodithuwakku et al. (2023)

**Abbreviations:** *LOD* limit of detection, *ISE* ion-selective electrode, *IC* ion chromatography, *HPLC* high-performance liquid chromatography, *GC* gas chromatography, *DGT* diffusive gradients in thin films

No analytical technique is universally suitable for all monitoring purposes. Laboratory methods provide the highest analytical accuracy but lack temporal resolution, whereas online sensors enable continuous process control at the expense of higher maintenance and calibration requirements. Consequently, monitoring strategy should be selected according to the primary objective, whether regulatory compliance, environmental assessment, or real-time process control.

## Future perspectives

The future of ammonia management is expected to extend beyond conventional pollutant removal towards integrated resource recovery within the framework of the circular economy. Rather than treating ammonia solely as an environmental contaminant, increasing attention is being directed towards its recovery and reuse as a valuable feedstock for fertilizer production and other industrial applications. Future wastewater treatment plants are therefore expected to evolve from pollution control facilities into resource recovery systems capable of simultaneously recovering nutrients, producing renewable energy, and minimizing environmental impacts. Rather than relying on a single treatment technology, future ammonia management will increasingly employ hybrid systems integrating biological, physicochemical, and membrane-based processes, with the optimal treatment strategy depending on wastewater characteristics, operational conditions, economic feasibility, and the desired recovery product. However, successful implementation of these technologies will require not only further technological development but also harmonized legislation, market readiness of recovered products, and comprehensive techno-economic and life-cycle assessments (Farghali et al., 2024; Śniatała et al., 2024).

Future advances in ammonia management are ex-pected to focus on the integration of complementary treatment technologies rather than further optimization of individual processes. Although significant progress has been achieved in biological, physicochemical, membrane-based, and electrochemical treatment methods, as discussed throughout this review, none of these technologies can simultaneously achieve high ammonia removal, nutrient recovery, energy efficiency, and economic feasibility under all operating conditions. Consequently, hybrid treatment systems have attracted increasing attention because they combine the strengths of individual technologies while minimizing their inherent limitations, allowing treatment configurations to be tailored to specific wastewater characteristics, operational requirements, and recovery objectives. Recent studies indicate that integrated membrane–biological systems, membrane contactors coupled with acid absorption, and biological nutrient removal combined with struvite precipitation represent particularly promising approaches for improving ammonia recovery and overall process sustainability. However, further full-scale validation together with comprehensive techno-economic and life-cycle assessments will be essential before these integrated systems can be widely implemented in municipal and industrial wastewater treatment facilities (Farghali et al., 2024).

The increasing complexity of wastewater treatment plants has stimulated the development of advanced digital tools for process monitoring and control. Data-driven control utilizes continuous measurements from online sensors together with historical operational data to optimize treatment performance, improve energy efficiency, and support operational decision-making in real time. For ammonia management specifically, continuous measurements from online ammonium or ammonia sensors can provide real-time input data for data-driven models and process control algorithms, enabling operational parameters such as aeration, pH, or hydraulic conditions to be adjusted in response to changes in ammonia concentration. One of the most advanced concepts is the digital twin, which represents a continuously updated virtual model of a physical wastewater treatment plant by integrating mechanistic process models with real-time monitoring data. Digital twins enable process simulation, predictive control, fault detection, and evaluation of different operational scenarios without interfering with the actual treatment process. Although digital twins and data-driven control have shown considerable potential for optimizing wastewater treatment processes, their current application is largely limited to individual research studies addressing specific operational challenges. Future implementation will require the integration of reliable online monitoring, robust process models, standardized data exchange, and advanced decision-support systems into a unified operational framework capable of supporting routine wastewater treatment plant management (Wang et al., 2024).

Future ammonia treatment technologies should be evaluated using comprehensive sustainability criteria rather than solely based on ammonia removal or nutrient recovery efficiency. In addition to treatment performance, life-cycle environmental impacts, energy demand, greenhouse gas emissions, and economic feasibility should be considered to identify the most sustainable solution for a given application (Pausta et al., 2024).

Ultimately, future ammonia management will depend not only on technological innovation but also on the successful integration of resource recovery, digitization, environmental sustainability, and regulatory requirements. Such an integrated approach will be essential for selecting the most appropriate treatment strategy for specific wastewater characteristics while supporting the transition towards a circular economy.

## Conclusion

This review highlights ammonia as a multifaceted pollutant whose environmental impact is governed not only by concentration but also by speciation, temporal variability, and local conditions. Regulatory pressure and the high resource value of ammonia jointly drive the development of efficient separation and recovery technologies.

Physicochemical processes such as stripping, membrane contactors, adsorption, and precipitation have demonstrated high removal efficiencies, particularly for concentrated waste streams, while biological pathways remain essential but are associated with greenhouse gas emissions and operational sensitivity. Upstream measures, including controlled-release fertilizers and urease or nitrification inhibitors, represent an effective preventive strategy by reducing peak ammonia emissions and complementing downstream treatment.

Accurate ammonia management requires appropriate analytical tools; thus, the selection of monitoring techniques should reflect concentration levels, matrix complexity, and temporal dynamics. Beyond conventional nutrient recovery, the utilization of recovered ammonia for $\text {CO}_2$ capture illustrates the potential for cross-sectoral integration between wastewater treatment and carbon management within the circular economy. Overall, future progress in ammonia control will rely on integrated approaches that combine source prevention, advanced separation technologies, and robust monitoring to minimize environmental impacts while enabling resource recovery.

## Data Availability

No datasets were generated or analysed during the current study.
